# Influence of acquisition parameters on MV‐CBCT image quality

**DOI:** 10.1120/jacmp.v13i1.3638

**Published:** 2012-01-05

**Authors:** Olivier Gayou

**Affiliations:** ^1^ Department of Radiation Oncology Allegheny General Hospital Pittsburgh PA 15212 USA

**Keywords:** image quality, MV‐CBCT, system performance

## Abstract

The production of high quality pretreatment images plays an increasing role in image‐guided radiotherapy (IGRT) and adaptive radiation therapy (ART). Megavoltage cone‐beam computed tomography (MV‐CBCT) is the simplest solution of all the commercially available volumetric imaging systems for localization. It also suffers the most from relatively poor contrast due to the energy range of the imaging photons. Several avenues can be investigated to improve MV‐CBCT image quality while maintaining an acceptable patient exposure: beam generation, detector technology, reconstruction parameters, and acquisition parameters. This article presents a study of the effects of the acquisition scan length and number of projections of a Siemens Artiste MV‐CBCT system on image quality within the range provided by the manufacturer. It also discusses other aspects not related to image quality one should consider when selecting an acquisition protocol. Noise and uniformity were measured on the image of a cylindrical water phantom. Spatial resolution was measured using the same phantom half filled with water to provide a sharp water/air interface to derive the modulation transfer function (MTF). Contrast‐to‐noise ratio (CNR) was measured on a pelvis‐shaped phantom with four inserts of different electron densities relative to water (1.043, 1.117, 1.513, and 0.459). Uniformity was independent of acquisition protocol. Noise decreased from 1.96% to 1.64% when the total number of projections was increased from 100 to 600 for a total exposure of 13.5 MU. The CNR showed a∓5% dependence on the number of projections and 10% dependence on the scan length. However, these variations were not statistically significant. The spatial resolution was unaffected by the arc length or the sampling rate. Acquisition parameters have little to no effect on the image quality of the MV‐CBCT system within the range of parameters available on the system. Considerations other than image quality, such as memory storage, acquisition speed, and individual projection image quality, speak in favor of the use of a coarse sampling rate on the short scan.

PACS numbers: 87.57.C‐; 87.57.nf

## I. INTRODUCTION

Image guidance (IG) using radiographic films and portal imagers has played an important role in patient positioning for radiation therapy (RT) for several decades.^(^ ^1^ ^)^ In the past few years, the presentation of volumetric image datasets in a sliced computed tomography (CT)‐like fashion has allowed for more accurate registration with the image actually used for planning. More frequent and more accurate imaging leads to the reduction of margins between the planning target volume (PTV) and the clinical target volume (CTV),^(^ ^2^ ^,^ ^3^ ^)^ allowing for dose escalation while maintaining sparing of healthy tissue. Furthermore, this development of online three‐dimensional (3D) imaging techniques offers new possibilities beyond localization. Structure segmentation and dose calculations can be performed on these images as well, opening the way for adaptive radiotherapy (ART).^(^ ^4^ ^–^ ^6^ ^)^

All major manufacturers of radiation therapy units have developed their own 3D‐imaging capability, based on different technology. In‐room kilovoltage (kV) CT on rails (CTVision, Siemens Medical Solutions, Concord, CA) relies on a conventional CT unit inside the treatment room and a transfer of the patient between the CT to the treatment unit.^(^ ^7^ ^–^ ^11^ ^)^ Megavoltage (MV) helical CT (CTrue, Tomotherapy, Madison, WI) is a MV fan‐beam system mounted on the TomoTherapy helical treatment unit.^(^ ^12^ ^–^ ^16^ ^)^ In kV cone‐beam CT (CBCT), an X‐ray tube is mounted on the treatment unit gantry at 90° from the megavoltage (MV) source and a series of two‐dimensional portal images acquired on the opposite electronic portal imaging device (EPID) is reconstructed into axial slices of a 3D volume (XVI, Elekta Oncology Systems, Norcross, GA, and OBI, Varian Medical System, Palo Alto, CA).^(^ ^17^ ^–^ ^23^ ^)^ The principle of MV‐CBCT is the same as that of kV‐CBCT, except that the X‐ray source is the 6 MV treatment beam itself, and the detector is an EPID optimized for MV photon detection (MVision, Siemens Medical Solutions, Concord, CA).^(^ ^24^ ^–^ ^27^ ^)^

The dosimetric aspects of cone‐beam imaging have been extensively studied, both for kV‐CBCT^(^ ^28^ ^–^ ^31^ ^)^ and MV‐CBCT.^(^ ^32^ ^–^ ^34^ ^)^ One of the shortcomings of MV‐CBCT is that due to the small yield of low‐energy photons, smaller differences in attenuation coefficients and reduced detection efficiency of the EPID lead to the need to increase exposure to enhance contrast, thereby increasing patient dose. However the MV energy range of the imaging beam allows for the dose to be easily modeled in a treatment planning system, and techniques have been developed to incorporate it in the treatment plan.^(^ ^34^ ^,^ ^35^ ^)^

Following pioneering work from more than 20 years ago,^(^ ^36^ ^,^ ^37^ ^)^ Siemens recently developed a new megavoltage imaging system, called the imaging beam line (IBL).^(^ ^38^ ^,^ ^39^ ^)^ For imaging purposes, the tungsten target is replaced with a low‐Z carbon target. Combined with a lower accelerating potential and removal of the flattening filter to lower the average photon energy, this new device allows for an improvement in image quality while keeping the same imaging dose. While the average beam energy is lower for the IBL than for the conventional MV‐CBCT, it is still in the MV range, and can be easily modeled for incorporation into a treatment plan.^(^ ^40^ ^)^

Morin et al.^(^ ^41^ ^)^ recently performed MV‐CBCT image optimization by changing various system parameters such as exposure, craniocaudal imaging length, voxel size, and slice thickness. The effect of reconstruction parameters, including binning, averaging and diffusion filtering of raw projections, as well as three different projection filters, was also examined. The study, performed with the conventional treatment beam line (TBL) MV‐CBCT on a Primus and an Oncor linear accelerator (Siemens Medical Solutions, Concord, CA), concluded that for optimized image quality, 512×512 transverse slices and 1 mm slice thickness should be used for reconstruction, and displayed with a 3 or 5 mm multiplanar reconstruction thickness, where slices are binned and averaged over a given thickness before display to reduce noise. It also showed that the use of a diffusion filter increased the contrast‐to‐noise ratio (CNR) by at least 30% for an exposure of 9 MU.

In this first‐generation MV‐CBCT system, some acquisition parameters were fixed by the manufacturer. In particular, the scan was performed over a 200° arc, starting at a gantry position of 270° and stopping at 110°. The sampling rate was 1° per projection, and the volume was reconstructed from 200 projections. Cone‐beam images are then reconstructed using the Feldkamp‐David‐Kress (FDK) algorithm.^(^ ^42^ ^)^ The latest generation of linear accelerators (linac) from Siemens Medical Solutions, the Artiste, offers a version of the MV‐CBCT software that allows the user to modify these acquisition parameters. First, the 200° arc option (the “short scan”) can be started from any position as long as the gantry does not cross the 180° line during the scan. Second, a “full scan”, covering 360° from 180° to 180°, can also be used. Scans can be performed clockwise or counterclockwise. The sampling rate can also be varied by choosing the number of projections used to reconstruct the image, from 100 to 400 projections for the short scan and from 180 to 600 projections for the full scan. When the sampling rate is changed for a given exposure protocol, the total exposure remains the same and the MU/projection is adjusted accordingly. In the work presented here, results of a comprehensive analysis of the impact of scan length and sampling rate on image quality using the IBL are presented. Using a variety of phantoms, contrast‐to‐noise ratio, MTF, uniformity, and noise calculations were performed in order to assess whether the limited range of acquisition parameters made available by the manufacturer had a clinically significant impact on image quality.

## II. MATERIALS AND METHODS

### A. Beam characteristics

All measurements were performed on a Siemens Artiste linear accelerator (Siemens Medical Solutions, Concord, CA), using a prerelease version of the IBL MV‐CBCT system. Compared to the conventional TBL system, the IBL uses lower photon beam energy in order to increase image quality. The average photon energy is reduced in three steps:^(^ ^38^ ^)^ (1) the electron beam energy is reduced from 6.0 MeV to 4.2 MeV; (2) the tungsten target is replaced by a carbon target, in which low‐energy photons are less attenuated due to the lower atomic number (Z=6 vs. 73); and (3) the flattening filter is removed, which also prevents the attenuation of low‐energy X‐rays. In addition, the flattening filter is a source of extrafocal radiation, which is a detriment to image quality. Unlike the method described in Faddegon et al.,^(^ ^38^ ^)^ the Artiste version of the IBL is calibrated like any other MV beam to deliver 1 cGy/MU at a depth of maximum dose for a $10\;\times \;10\;{\text{cm}}^{2}$ field at an source to axis distance of 100 cm.

During MV‐CBCT, the gantry rotates around the patient and at given gantry positions, the EPID data acquisition system is triggered and an X‐ray image is taken. The flat panel detector consists of 1024×1024 amorphous silicon (a‐Si) photodiodes connected to thin film transistors. The diodes are placed every 400 μm in both directions, for an active area of $409.6\;\times \;409.6\;{\text{mm}}^{2}$. The depth for each pixel is 16 bit and the shortest frame readout period is 285 ms. In this study, three parameters were varied: the arc length, the sampling rate (number of degree per projection), and the total exposure. Nine protocols were created and are summarized in Table 1. All images were taken with a cross‐plane field size of 27.4 cm, and an in‐plane field size adjusted to match the phantom that was being imaged. All datasets were reconstructed according to the recommendations of Morin et al.^(^ ^41^ ^)^ Even though these parameters were optimized for the TBL version of MV‐CBCT rather than the IBL version, which has a different energy spectrum, they produced high‐quality images useful for comparison of the acquisition parameters. For each dataset, 512×512 transverse slices with a 1 mm slice thickness were created with the “smoothing” band‐pass filter. No diffusion filter was used, as it was not available at the time. The transverse slices were binned and averaged over a thickness of 4.8 mm for display and analysis. Uniformity, noise, contrast‐to‐noise ratio, and spatial resolution were analyzed using MATLAB (The MathWorks Inc., Natick, MA).

**Table 1:** MV‐CBCT acquisition protocols used in this study: scan length, sampling rate, and total exposure.

*Protocol Number*	*Start*	*Arc Stop*	*Length*	*Number of Projections*	*Sampling Rate Degree/Projection*	*Exposure (MU)*	*MU/ Projection*
1	180°	180°	360°	180	2.0	13.5	0.0750
2	180°	180°	360°	360	1.0	13.5	0.0375
3	180°	180°	360°	400	0.9	13.5	0.0338
4	180°	180°	360°	450	0.8	13.5	0.0300
5	180°	180°	360°	600	0.6	13.5	0.0225
6	270°	110°	200°	100	2.0	13.5	0.1350
7	270°	110°	200°	200	1.0	13.5	0.0675
8	270°	110°	200°	400	0.5	13.5	0.0338
9	180°	180°	360°	360	1.0	7.2	0.0200

### B. Noise and uniformity

**Figure 1:**
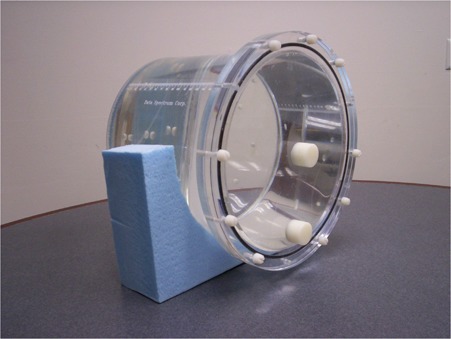
Cylindrical water phantom used for noise and uniformity measurements. The same phantom half filled with water was used to determine the modulation transfer function.

A 22 cm diameter, 17 cm height cylindrical phantom filled with water was used, as shown in Fig. 1. The CBCT images were acquired with a fully open field of $27.4\;\times \;27.4\;{\text{cm}}^{2}$ and reconstructed using the “smoothing pelvis” filter. Four regions of interest (ROI) were identified: a 2.5 cm diameter circular central region (${\text{ROI}}_{\text{center}}$), a 2 cm thick ring at the periphery (${\text{ROI}}_{\text{periphery}}$), a 19 cm diameter circular region encompassing most of the water inside the cylinder (${\text{ROI}}_{\text{water}}$), and a 2 cm thick ring outside the phantom (${\text{ROI}}_{\text{air}}$). The uniformity was then defined as the difference between the mean pixel values in the central and peripheral regions, normalized to the difference of mean pixel value in the water and air regions:^(^ ^41^ ^)^

(1) $$Uniformity=\left[1-\frac{\left|mean(ROI_{center})-mean(ROI_{peripheral})\right|}{mean(ROI_{water})-Mean(ROI_{air})}\right]\times 100$$

The noise was defined as the standard deviation of the water ROI normalized to the difference of mean pixel value in the water and air regions:

(2) $$Noise=\left[\frac{stdev(ROI_{water})}{mean(ROI_{water})-mean(ROI_{air})}\right]\times 100$$

Both uniformity and noise were measured and averaged over six consecutive transverse slices.

### C. Contrast‐to‐noise ratio

**Figure 2:**
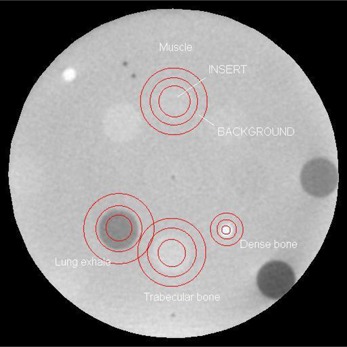
Regions of interest contoured on phantom image used for contrast‐to‐noise ratio calculation: the circular signal and the ring‐shaped background are centered in each insert of different electron density.

Contrast‐to‐noise ratio was measured in a pelvis‐shaped (in the transverse direction) phantom (CIRS Model 62, Norfolk, VA). The thickness of the phantom was 5 cm in the craniocaudal direction; therefore, a $27.4\;\times 7\;{\text{cm}}^{2}$ field size was used for CB acquisition. Volumetric images were reconstructed with the “smoothing pelvis” filter. Eight 3 cm diameter plugs of different electron density were inserted in a 12 cm diameter pattern around the center of the phantom. Four of these inserts were analyzed in this study: muscle (electron density relative to water rED=1.043), trabecular bone (rED=1.117), dense bone (rED=1.513), and lung in the exhale phase (rED=0.459). For each insert, a 2.5 cm diameter circular region (${\text{ROI}}_{\text{insert}}$) was contoured on the CB image (a 0.8 cm diameter ROI was used for the dense bone region due to the smaller insert). A 1 cm thick ring‐shaped background region (${\text{ROI}}_{\text{background}}$) was also contoured around each insert, as illustrated in Fig. 2. The mean pixel value for each insert and background ROI was then calculated along with its standard deviation. The CNR was then calculated according to:

(3) $$CNR=\frac{\left|mean(ROI_{insert})-mean(ROI_{background})\right|}{1{/}_{2}\left[stdev(ROI_{insert})+stdev(ROI_{background})\right]}$$

The measurement was repeated and averaged over six consecutive transverse slices.

### D. Spatial resolution

**Figure 3:**
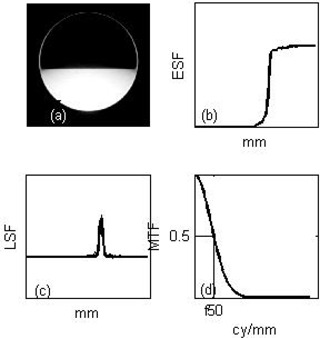
Processing chain to obtain the modulation transfer function (MTF) and frequency at 50% contrast ($f_{50}$) from the raw image of a half‐filled water phantom: (a) the image of the half‐filled water cylindrical phantom is acquired; (b) the edge spread function (ESF) at the air/water interface is obtained; (c) the line spread function (LSF) is calculated as the gradient of the ESF and fitted with a Gaussian to remove low‐level noise; (d) a Fourier‐transform of the LSF yields the MTF.

The cylindrical water phantom described above and shown in Fig. 1 was half filled with water to provide a sharp water/air interface. The edge spread function (ESF) was defined as the pixel values along a line perpendicular to the interface. The line spread function (LSF) was then defined as the first derivative of the ESF, fitted by a Gaussian function to remove the effect of the noise at the interface.^(^ ^43^ ^)^ Finally, the modulation transfer function (MTF) was calculated as the fast Fourier transform of the Gaussian fit of the LSF. The frequency at 50% contrast ($f_{50}$) was extracted from the MTF and compared between different imaging protocols. The $f_{50}$ measurement was repeated and averaged over six consecutive axial slices. The process chain to obtain the MTF and $f_{50}$ from the raw image is illustrated in Fig. 3(a)–3(d).

## III. RESULTS

### A. Noise and uniformity

**Figure 4:**
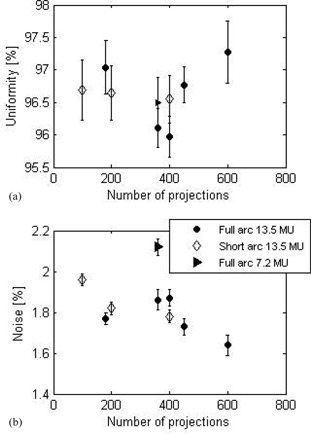
Uniformity (a) and noise (b) as a function of the total number of projections, measured on the full water phantom. Error bars correspond to one standard deviation of the measurements taken over six consecutive slices.

Noise and uniformity results are shown on Fig. 4. While uniformity is independent of acquisition protocol, noise appears to have a weak dependence on the total number of projections. For a given exposure of 13.5 MU, noise decreases from 1.96% to 1.64% as the total number of projections increases from 100 to 600, regardless of the arc length. As expected, noise increases as exposure decreases: for 360 projections, noise increases from 1.86% to 2.12% as the exposure decreases from 13.5 to 7.2 MU.

### B. Contrast‐to‐noise ratio

**Figure 5:**
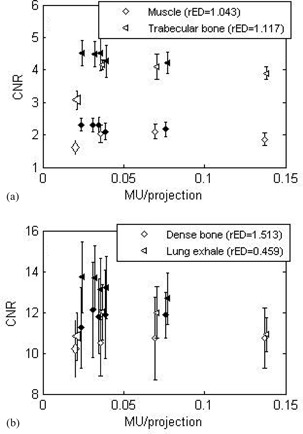
Contrast‐to‐noise ratio as a function of the MU/projection, for the full arc (empty symbols) and the short arc (solid symbols) for: (a) low‐contrast inserts and (b) high‐contrast inserts, measured on the CIRS phantom. The low MU protocol is displayed as the large open symbols. Error bars correspond to one standard deviation of the measurements taken over six consecutive slices.

Contrast‐to‐noise ratio results are presented in Fig. 5(a) for the low‐contrast inserts (muscle, rED=1.043; trabecular bone, rED=1.117), and Fig. 5(b) for the high‐contrast inserts (dense bone, rED=1.513; lung exhale, rED=0.459) as a function of MU per projection. A slight decrease in CNR was observed as the MU per projection increased. The CNR was, on average, approximately 10% lower for the short arc than for the full scan. However, for each ROI, the CNR was averaged over six consecutive 5 mm thick slices. The standard deviation over these six measurements was approximately 20% for all inserts and all protocols. Therefore, the variation of CNR as a function of acquisition protocol bears no statistical significance. The CNR for the low exposure protocol (7.2 MU total, 360 projections, 0.02 MU/projection) was, on average, 25% lower than that of the higher exposure protocol with a similar MU per projection (13.5 MU total, 600 projections, 0.0225 MU/projection).

### C. Spatial resolution

**Figure 6:**
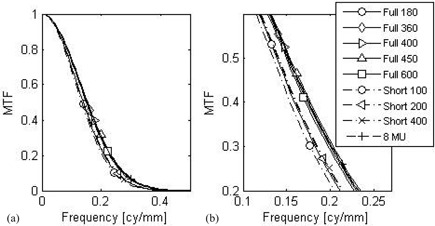
Modulation transfer function (a) for each protocol, measured on the half water phantom; (b) zoom around the 50% contrast region of the MTF for clarity.

**Figure 7:**
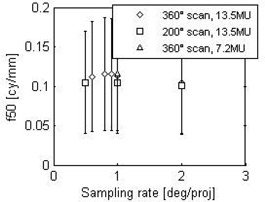
Frequency at 50% contrast ($f_{50}$) as a function of the sampling rate. Error bars correspond to one standard deviation of the measurements taken over six consecutive slices.

The effect of arc length and number of projections on the spatial resolution is illustrated in Fig. 6, showing that the MTFs for each acquisition protocol are close to each other. The frequency at 50% contrast $f_{50}$ is plotted as a function of the sampling rate (Fig. 7). As expected, the spatial resolution is independent of exposure, as shown by the similar $f_{50}$ for the 13.5 and 7.2 MU protocols with identical arcs. The spatial resolution appears unaffected by the arc length or the sampling rate.

## IV. DISCUSSION

Objective measurements of image quality of megavoltage cone‐beam computed tomography were compared for different acquisition protocols, where the arc length, the total number of projections, and total exposure were varied. The effects of these parameters on noise, uniformity, contrast‐to‐noise, and spatial resolution were studied. The main result from this study is that none of these measures were affected by the arc length or the total number of projections. However, the total exposure for a constant number of projections had the expected effect on noise and CNR, as reported earlier,^(^ ^33^ ^,^ ^41^ ^)^ where the CNR varied as the inverse of the square root of the dose.

The use of the edge spread function of a water/air interface to determine spatial resolution differs from the method used by Morin et al.^(^ ^41^ ^)^ of a direct measurement of the point spread function using a brass wire. In particular, the additional step of differentiating the ESF to obtain the LSF introduces noise to the process. However, the sharpness of the interface eliminates the need to deconvolve the thickness of the wire from the ESF. Furthermore, the use of the ESF defines the spatial resolution only in one direction, perpendicular to the edge. However, it is expected that spatial resolution is direction‐independent, due to the circular nature of the CBCT scan. In the end, the actual method used to define spatial resolution is not important to this study because only relative measurements are used to compare different acquisition protocols. The results show that the acquisition protocol has no effect on spatial resolution within the range of protocols allowed by the system. It has been shown that 20–40 X‐ray projections provided image quality sufficient for localization in the case of kV‐CBCT, albeit with a specialized reconstruction algorithm different from the FDK algorithm.^(^ ^44^ ^)^ However, within the range of values allowed by the manufacturer, which includes a minimum of 100 projections, the spatial resolution is limited by that of the flat‐panel detector, and no improvement is observed by increasing the sampling rate.

It should be noted that there is another acquisition parameter that affects image quality: the cone angle, defined by the in‐plane field size. While the out‐of‐plane field size is fixed at 27.4 cm by the manufacturer, the in‐plane field size is adjustable up to 27.4 cm. As smaller fields produce less scatter, the image quality should improve as the jaws are closed. This effect was studied for the TBL, showing that the CNR was increased by 20% when the field size decreased from 27.4 to 5.0 cm.^(^ ^26^ ^)^ However, that parameter was not included in the study because image quality is not an aspect that is taken into account in its selection. Typically at our institution, the smallest field size that covers all the organs of interest on the image is selected in order to reduce the unnecessary normal tissue exposure.

The choice of acquisition parameters can be driven by considerations other than the quality of the reconstructed cone‐beam image. For example, the use of a higher number of projections will increase the acquisition time since the gantry moves slower to allow for completion of detector readout before the next projection angle is reached. For a 10 MU acquisition protocol with 200° arc length, acquiring one image every degree for a total of 200 images leads to a gantry speed of 166°/min, corresponding to a total acquisition time of 72.3 sec. Acquiring one image every 2° for a total of 100 images leads to a gantry speed of 221°/min, corresponding to a total acquisition time of 54.3 sec. It is useful to note that these acquisition times measured with the carbon target of the IBL are longer than the typical acquisition times encountered with the conventional MV‐CBCT. This is due to a lower nominal dose rate for cone beam related to the lower efficiency of the carbon target. Likewise, the reconstruction time for the CB dataset is proportional to the number of projections. Therefore, minimizing the number of projections optimizes patient throughput when most patients are imaged daily on a given linac. In addition, increasing the number of projections increases the memory required for storing the projections, if they are required for research purposes.^(^ ^45^ ^)^ Coded with 16‐bit accuracy, the size of the projection file for the 1012×1012 active pixels detector panel is 204 MB for 100 projections, 409 MB for 200 projections, and 738 MB for 360 projections. Finally, for some lung cases, the couch lateral position can be quite far away from zero, and there is a risk of collision with the flat panel during CB acquisition. For these cases, it is preferable to set the arc so that the detector remains on the contralateral side, prohibiting a full arc acquisition.

While the sampling rate has little to no effect on the quality of the reconstructed image, it does affect the quality of the individual projection images by increasing the MU/projection. At our institution, prior to the first lung SBRT fraction for each patient, we review the individual projections displayed in a movie to ensure that tumor motion is consistent with what was observed at the time of the 4D‐CT acquisition.^(^ ^46^ ^)^ It allows us to check that the ITV to PTV margins are appropriate. Doubling the MU used for each projection by using the 100 projection instead of the 200 projection protocol helped us distinguish the tumor inside the lung with more ease.

**Figure 8:**
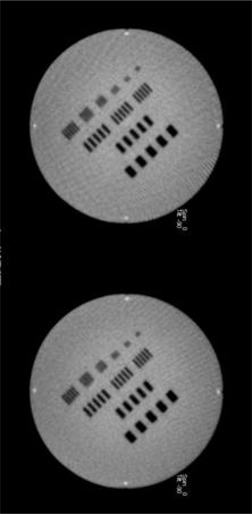
IBL MV‐CBCT images of an image quality phantom acquired with the short scan, with 100 projections (left) and 200 projections (right).

The above‐mentioned speed of acquisition, memory storage, and higher individual projection quality are all in favor of coarse‐resolution projection sampling, and demonstrate the usefulness of being able to move away from the set acquisition parameters available on the Primus or Oncor linacs (200° arc, start at 270°, sampling rate of 1° per projection). Figure 8 shows images of the image quality phantom with 100 projections (left) versus 200 projections (right). Lowering the sampling rate did not introduce unacceptable artifacts, even in the high‐contrast regions around the bar patterns. It would be interesting to know the breaking point beyond 2° per projection where image quality is clinically significantly degraded. Unfortunately, the clinical system used in this study did not allow us to extend the parameters outside the available range. A kV‐CBCT study showed that below one‐sixth the number of projections used for a full scan, artifacts and contrast become so severe that accurate registration is no longer possible.^(^ ^47^ ^)^ This corresponds to a sampling rate of 6° per projection, which is outside the available range for this study.

Whether the image is used for diagnosis, registration, or segmentation, the notion of image quality is inherently subjective. The practicality of megavoltage cone‐beam CT for localization purposes has been demonstrated when registration accuracy on the order of 1–2 mm is required.^(^ ^27^ ^,^ ^48^ ^,^ ^49^ ^)^ Improvements in image quality can be helpful if MV‐CBCT images are to be used for segmentation in an adaptive radiotherapy setting, where the daily pretreatment image is used to adapt the plan, whether offline or online. The present study shows that little improvement can be achieved from acquisition protocols within the range offered by the manufacturer. However, technological developments as demonstrated by the transition from TBL to IBL, as well as the application of the optimal reconstruction parameters and filters,^(^ ^41^ ^)^ are very promising.

## V. CONCLUSIONS

The effects of scan length and number of projections on megavoltage cone‐beam CT image quality were studied. Quantitative image quality measures such as noise, uniformity, contrast‐to‐noise ratio, and spatial resolution were calculated. The results show that the CB acquisition parameters have little to no effect on image quality within the range allowed by the manufacturer. By contrast, the effects of reconstruction parameters such as image thickness, pixel size, and reconstruction filters are a much more promising avenue to pursue to improve image quality. However, considerations other than image quality — such as memory storage, acquisition speed, and individual projection image quality — point to the preferential use of a coarse sampling rate on the short scan.

## ACKNOWLEDGMENTS

This work was partially funded by Siemens Medical Solutions.
